# The correlation between selenium intake and lung function in asthmatic people: a cross-sectional study

**DOI:** 10.3389/fnut.2024.1362119

**Published:** 2024-05-17

**Authors:** Hejun Jiang, Guijun Yang, Jing Chen, Shuhua Yuan, Jinhong Wu, Jing Zhang, Lei Zhang, Jiajun Yuan, Jilei Lin, Jiande Chen, Yong Yin

**Affiliations:** ^1^Department of Respiratory Medicine, Shanghai Children’s Medical Center, School of Medicine, Shanghai Jiao Tong University, Shanghai, China; ^2^Department of Respiratory Medicine, Linyi Maternal and Child Healthcare Hospital, Linyi Branch of Shanghai Children’s Medical Center, Shanghai JiaoTong University School of Medicine, Linyi, Shandong, China; ^3^Medical Department of Shanghai Children’s Medical Center, Shanghai Jiao Tong University School of Medicine, Shanghai, China; ^4^Pediatric AI Clinical Application and Research Center, Shanghai Children’s Medical Center, Shanghai, China; ^5^Shanghai Engineering Research Center of Intelligence Pediatrics (SERCIP), Shanghai, China; ^6^Shanghai Children’s Medical Center Pediatric Medical Complex (Pudong), Shanghai, China

**Keywords:** NHANES (National Health and nutrition examination survey), asthma, lung function, cross-sectional study, selenium intake

## Abstract

**Objective:**

This study aimed to examine the correlation between selenium intake and lung function in asthmatic people.

**Methods:**

A total of 4,541 individuals in the US National Health and Nutrition Examination Survey (NHANES) were included in this study. Multivariate linear regression, variance inflation factor, restricted cubic splines and quantile regression were used to analyze the relationship between Se intake and lung function. We divided selenium intake into four levels based on quartiles: Q1: Se ≤ 76.75 mcg/d; Q2: 76.75–105.1 mcg/d; Q3: 105.1–137.65 mcg/d; and Q4: Se ≥137.65 mcg/d.

**Results:**

Asthma was negatively associated with the Ratio of Forced Expiratory Volume 1st Second to Forced Vital Capacity (FEV1/FVC) (β = −0.04, 95% CI: −0.06 to −0.02) and FEV1 (β = −215, 95% CI: −340 to −90). Se intake was positively associated with Forced Expiratory Volume 1st Second (FEV1) (β =3.30 95% CI: 2.60 to 4.00) and Forced Vital Capacity (FVC) (β =4.30, 95% CI: 3.50 to 5.10). In asthmatic individuals, the positive effects of Se intake on FVC were enhanced with increasing Se intake, while the positive effects of Se intake on FEV1 varied less dramatically. High Se intake (Q4 level, above 137.65 mcg/d) improved FVC (β = 353, 95% CI: 80 to 626) and FEV1 (β = 543, 95% CI: 118 to 969) in asthmatic patients compared to low Se intake (Q1 level, below 76.75 mcg/d). At the Q2 level (76.75–105.1 mcg/d) and Q4 level (Se ≥137.65 mcg/d) of Se intake, the correlation between FEV1 and asthma disappeared.

**Conclusion:**

Our research has revealed a positive correlation between selenium intake and lung function in asthma patients and the strength of this positive correlation is related to the amount of selenium intake. We recommend that asthma patients consume 137.65 mcg to 200 mcg of selenium daily to improve pulmonary function while avoiding the adverse effects of selenium on the human body.

## 1 Introduction

Asthma is an important global noncommunicable disease that has significant public health consequences for children and adults, including a high incidence rate and mortality of serious cases (1). Asthmatic patients usually have poor lung function, especially those with poor asthma control. Studies have shown that this may be related to oxidative stress (2). The oxidative stress markers raised in asthmatics correlate to disease severity and phenotype (3). In the lung, the exhaled breath condensate of asthmatic patients showed higher levels of some oxidative stress biomarkers, such as H2O2 and NO (4, 5). In blood, blood leukocytes from asthmatic patients were shown to produce higher O2●- levels and contain lower GSH levels and higher blood levels of protein carbonyls, lipid peroxides and nitrites/nitrates. Moreover, lower glutathione peroxidase activity and GSH concentration were shown in asthmatic patients (6–8). Therefore, intervening in oxidative stress in asthma patients seems to be one of the means to improve their lung function.

As an essential trace element, selenium (Se) is a core component of human glutathione peroxidase and is involved in the regulation of a variety of metabolic processes, including cell protection against oxidative stress (9, 10), redox signaling (11), immune response (12), and thyroid hormone metabolism (13, 14). Diet is the primary source of Se (15). Se deficiency affects one-seventh of the world’s population (15) and is associated with an increased risk of cancer (16), neurodegenerative diseases (17–19), and thyroid dysfunction (20, 21). In animal experiments, oxidative stress induced by Se deficiency could lead to inflammation, apoptosis, necroptosis, and fibrosis in the lungs of mice and calves (22, 23). Experimental studies have shown that Se can control allergic mediators and symptoms in rhinitis and asthma and reduce pulmonary inflammation and airway mucus secretion, helping to open obstructed bronchi in mice (24).

However, thus far, epidemiological evidence about the protective role of Se on abnormal pulmonary function in asthma patients is still limited.

Therefore, this paper aims to explore the potential interaction between Se and asthma on lung function.

## 2 Methods

### 2.1 Data source

Our study analyzed data from the National Health and Nutrition Survey (NHANES), which collected information representative of the civilian noninstitutionalized population in the United States to investigate the prevalence of major diseases and identify risk factors for diseases. The NHANES study was approved by the National Center for Health Statistics Institutional Review Board, and all participants provided written informed consent. The data used in this study were limited to three consecutive cycles from 2007 to 2012 because we needed complete exposure and outcome data. We included individuals with complete information on the needed variables in these cycles.

**Figure 1:**
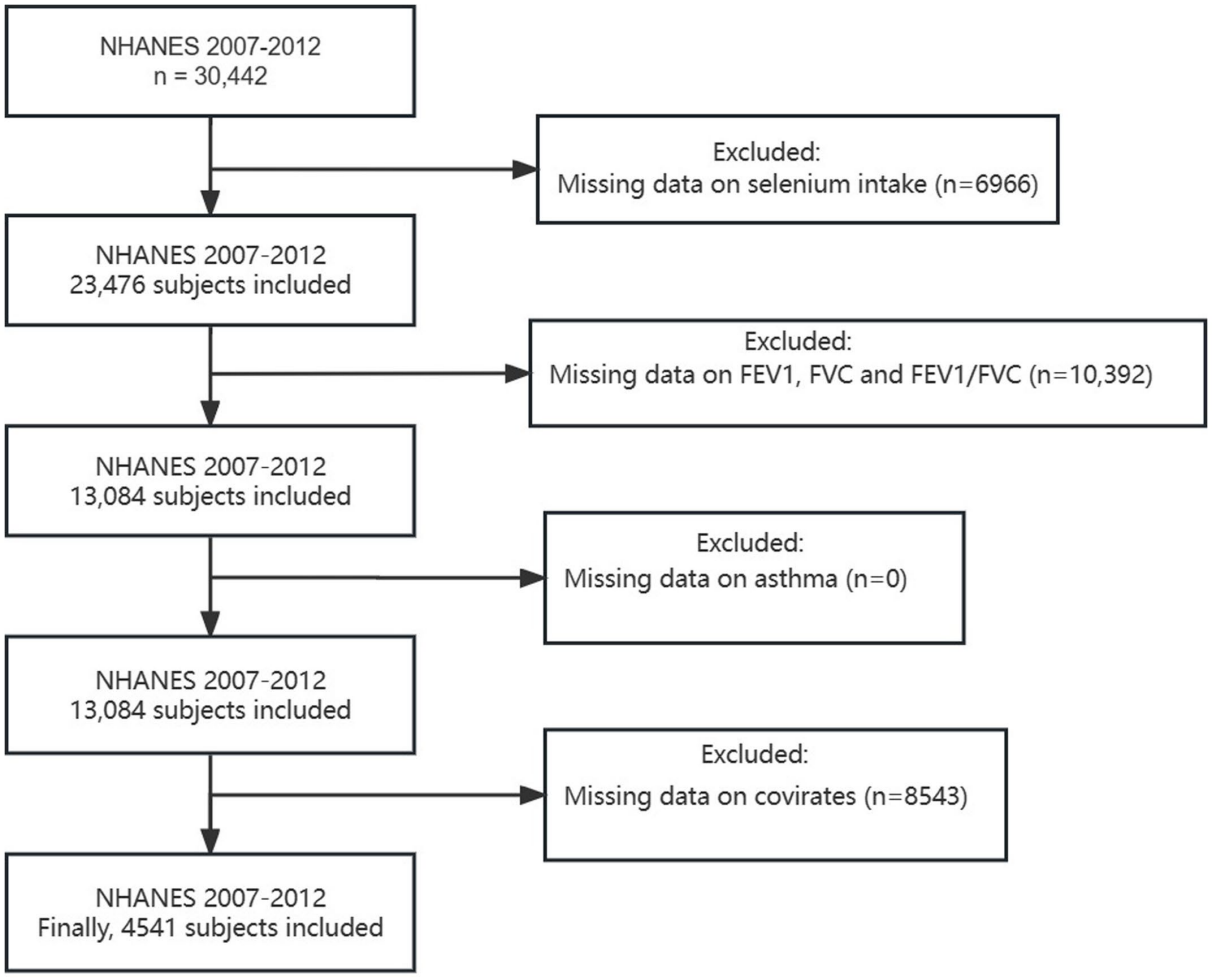
Flowchart of the sample selection from NHANES 2007–2012.

A total of 30,442 participants were included in the NHANES during 2007–2012. For this cross-sectional analysis, we excluded the following participants: (1) missing data on Se intake (*n* = 6,966), (2) unqualified data on lung function (specific standards can be found in Section 2.3) (*n* = 10,392), (3) incomplete data on asthma (*n* = 0), and (4) incomplete data on covariates (*n* = 8,543). As a result, 4,541 participants were eventually enrolled in the present study (Figure 1).

### 2.2 Se intake

In each NHANES cycle, participants provided detailed dietary intake information for two 24-h periods, which were then used to estimate intakes of energy, nutrients, and other food components. The first dietary recall was collected in person during the NHANES visit, while the second was collected by telephone 3 to 10 days later. For these analyses, total estimated Se intake (micrograms, mcg) was averaged over the two recall periods. Se levels were categorized into high (Se+) and low (Se−) according to the medians of the distribution (105.1 mcg).

### 2.3 Lung function

Survey participants aged 6 to 79 years were eligible for the spirometry component. Specific exclusion criteria that were employed for baseline spirometry included criteria employed in earlier NHANES spirometry surveys. Excluded from testing were examinees who had current chest pain or a physical problem with forceful expiration, were taking supplemental oxygen, had recent surgery of the eye, chest or abdomen, had a recent heart attack, stroke, tuberculosis exposure or had recently coughed up blood. Adults with a personal history of detached retina or a collapsed lung and children with painful ear infections were also excluded. Lung function was measured by Ohio 822/827 dry-rolling seal volume spirometers. Spirometry was performed in the standing position unless the participant was physically impaired. The participant was asked to elevate the chin and extend the neck slightly and place a nose clip on his or her nose during testing, the latter to prevent air leaks. He/she was then instructed to perform a series of maximal forced expiratory maneuvers. In each maneuver, the participant took the deepest breath possible to fill the lungs with air, then put the mouthpiece into his/her mouth making a tight seal and then blew the air out as hard and fast as possible. Participants aged 6–10 years were asked to blow out the air for a minimum of 3 s of exhalation, whereas those aged 11–79 years were asked to blow out the air for a minimum of 6 s of exhalation. We used forced vital capacity (FVC), forced expiratory volume 1st second (FEV1) and the ratio of FEV1 to FVC to analyze the effects of Se intake and asthma on pulmonary function.

### 2.4 With or without current asthma

Current physician-diagnosed asthma (hereafter referred to as “current asthma”) was defined by a positive answer to the following questions: “Do you still have asthma?.” If the respondent answered “yes” to this question, she/he was classified as a participant with current asthma. If not, s/he was classified as a participant without current asthma. The asthma-Se patterns were divided into four categories: without current asthma and low Se intake (Asthma− Se−), without current asthma and high Se intake (Asthma− Se+), with current asthma and low Se intake (Asthma+ Se−) and with current asthma and high Se intake (Asthma+ Se+).

### 2.5 Covariates

Based on previous studies related to lung function, we used the following variables as covariates (25). Sociodemographic characteristics were collected using the sample person and family demographics questionnaires, including age, sex, race, ratio of family income to poverty (PIR) and education level. We dichotomized educational attainment into three levels, including less than a high school degree, high school grade/GED or some college/AA degree and college graduate or above. Body mass index (BMI) was calculated as weight (kg) divided by the square of height in (m). Triglycerides in blood, hemoglobin, high-density cholesterol (HDL) and urinary creatinine were measured using the Roche Modular P chemistry analyzer. Smoking status was defined as never smokers (<100 cigarettes in a lifetime), ever smokers (>100 cigarettes in a lifetime, now not smoking at all), and current smokers (>100 cigarettes in a lifetime, some days or every day).

### 2.6 Statistical analysis

All statistical analyses were performed in R (version 4.3.1) software. Participants were characterized according to the quartiles of Se intake, with means (standard deviations) for continuous variables and percentages for categorical variables. Baseline characteristics were compared across Se intake quartiles using chi-square tests, ANOVA and Wilcoxon rank-sum tests depending on the nature of the data. To assess the association among asthma, Se intake and pulmonary function, we conducted univariate and multivariate linear regression models, including the unadjusted model (model 1) and fully adjusted model (model 2, age, race, PIR, education levels, triglycerides, smoking status, BMI, ASBP, ADBP, urinary creatinine, high-density cholesterol (HDL), and hemoglobin), to reduce the impact of confounding factors. The variance inflation factor (VIF) was used to test for multicollinearity between variables. To explore the role of Se intake in the relationship between asthma and pulmonary function, multivariable linear regressions and restricted cubic splines (from the R package “rms”) with four default knots based on sample size were performed across Se intake. We also used quantile regression to assess the relationship among asthma, Se intake and pulmonary function. Finally, we used linear regression to investigate whether the difference in lung function between participants with current asthma and participants without current asthma disappeared under different selenium intake levels.

## 3 Results

### 3.1 Baseline characteristics

Table 1 shows the baseline characteristics of the total population and participants by asthma status. Participants in the asthma+ group were more likely to be non-Hispanic but had high levels of hemoglobin. These participants also tended to have higher levels of Se intake. Most importantly, we observed that asthmatic participants had poor FEV1, FVC and FEV1/FVC. We found that all participants were older than 20 years. No other significant differences were observed in the results.

**Table 1:** Baseline characteristics stratified by asthma status.

Characteristic	*N* ^1^	Asthma			*p* Value^3^
		Overall, *N* = 4,541 (100%)^2^	Without current asthma *N* = 4,212 (92%)^2^	With current asthma *N* = 329 (8.0%)^2^	
Age (years)	4,541	45.0 (32.0, 57.0)	45.0 (32.0, 57.0)	43.0 (30.0, 58.0)	0.6
BMI (kg/m^2^)	4,541	28 (24, 32)	27 (24, 32)	29 (24, 34)	0.12
Race	4,541				**0.009**
Mexican American		728 (8.1%)	704 (8.5%)	24 (3.7%)	
Other Hispanic		467 (5.3%)	445 (5.4%)	22 (3.4%)	
Non-Hispanic		3,182 (83%)	2,912 (82%)	270 (89%)	
Other Race-Including Multi-Racia		164 (4.0%)	151 (4.1%)	13 (3.9%)	
Gender	4,541				0.089
Male		2,225 (49%)	2,099 (50%)	126 (43%)	
Female		2,316 (51%)	2,113 (50%)	203 (57%)	
Family income to poverty	4,541	3.13 (1.45, 5.00)	3.16 (1.51, 5.00)	2.48 (1.10, 4.97)	0.15
Education level	4,541				0.9
Less than high school degree		1,066 (15%)	997 (15%)	69 (17%)	
High school grad/GED or some college/AA degree		2,326 (52%)	2,151 (52%)	175 (50%)	
College graduate or above		1,149 (33%)	1,064 (33%)	85 (34%)	
Triglyceride (mmol/L)	4,541	1.22 (0.87, 1.73)	1.23 (0.86, 1.73)	1.21 (0.92, 1.73)	0.6
Smoke	4,541				0.091
Never smokers		2,516 (55%)	2,348 (55%)	168 (48%)	
Ever smokers		1,088 (25%)	1,009 (25%)	79 (25%)	
Current smokers		937 (20%)	855 (20%)	82 (27%)	
Hemoglobin (g/dL)	4,541	14.40 (13.50, 15.40)	14.40 (13.50, 15.40)	14.10 (13.10, 15.10)	**0.016**
Creatinine, urine (umol/L)	4,541	112 (65, 166)	113 (65, 166)	104 (61, 168)	0.5
HDL (mg/dL)	4,541	51 (43, 62)	51 (43, 62)	51 (40, 61)	0.3
ASBP (mmHg)	4,541	118 (110, 128)	118 (110, 128)	120 (110, 130)	0.5
ADBP (mmHg)	4,541	70 (64, 78)	70 (64, 78)	70 (62, 76)	0.3
FEV1	4,541	3,170 (2,591, 3,852)	3,192 (2,602, 3,870)	2,927 (2,438, 3,519)	**<0.001**
FVC	4,541	4,048 (3,356, 4,906)	4,064 (3,364, 4,928)	3,906 (3,147, 4,565)	**0.024**
FEV1/FVC	4,541	0.79 (0.74, 0.83)	0.79 (0.74, 0.83)	0.75 (0.69, 0.81)	**<0.001**
Se intake(mcg/d)	4,541	109 (81, 143)	110 (82, 143)	92 (71, 130)	**0.001**

1 N not Missing (unweighted).

2 Median (IQR) for continuous; *n* (%) for categorical.

3 Wilcoxon rank-sum test for complex survey samples; chi-squared test with Rao & Scott’s second-order correction. The bold values signify significant statistical differences (*p* < 0.05).

### 3.2 The differences in FEV1, FVC and FEV1/FVC in the patterns of asthma-Se

**Figure 2:**
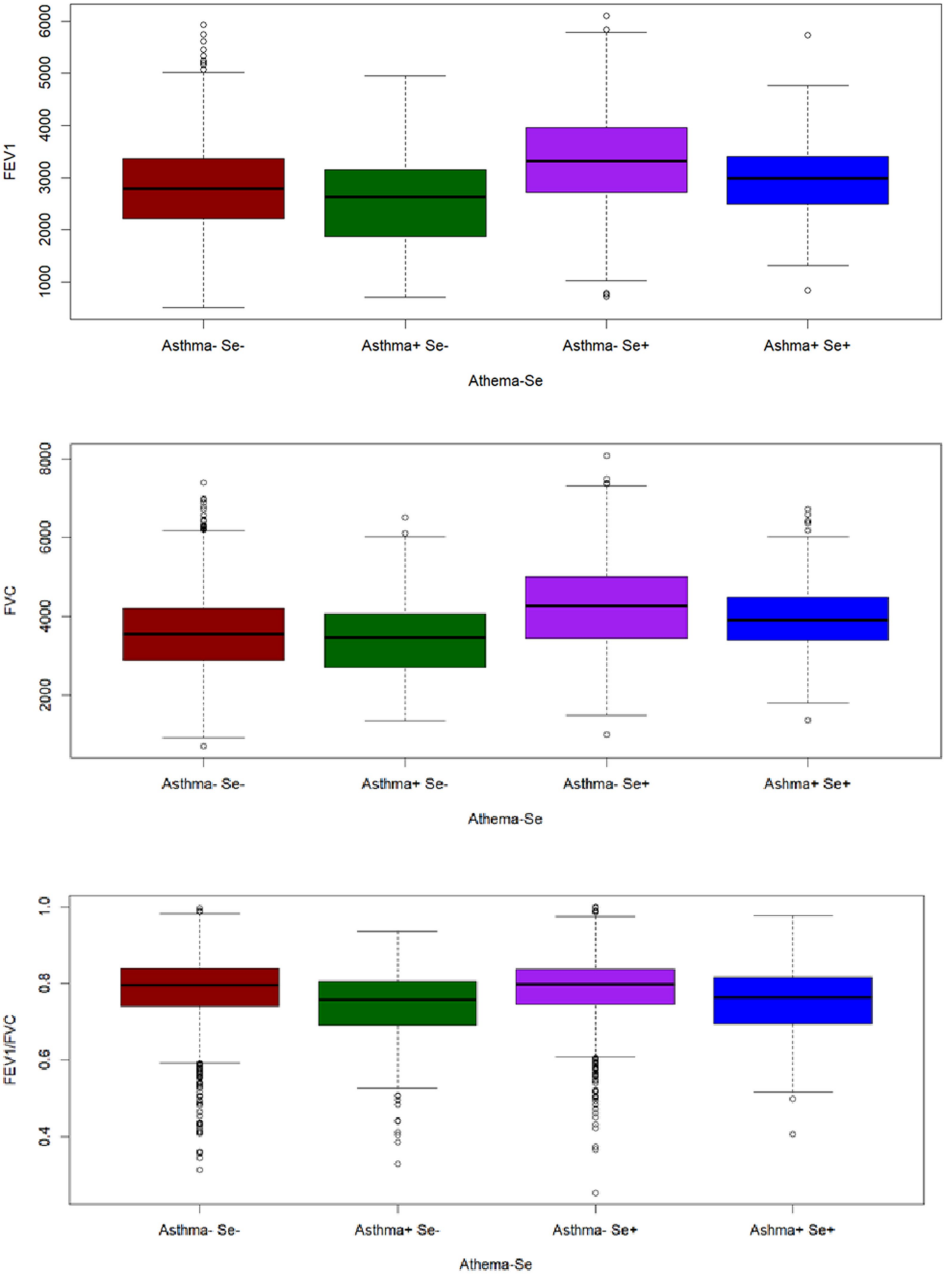
The distributions of lung function in different combinations of asthma and Se intake.

The differences in FEV1, FVC and FEV1/FVC in the patterns of asthma-Se are shown in Figure 2. The asthma-Se+ group had the highest FEV1, FVC and FEV1/FVC, whereas the lowest FEV1 and FVC was observed in the asthma+ Se− group. Furthermore, we found that the FEV1, FVC and FEV1/FVC in the asthma+ Se+ group were higher than those in the asthma+ Se-group. Asthma− Se + and asthma− Se − groups showed similar outcomes. All results are significant (*p* < 0.05).

### 3.3 Associations of Se intake and lung function in participants with different asthma statuses

In the unadjusted model (model 1), Se was positively associated with FEV1 (β = 6.30, 95% CI: 5.50 to 7.10) and FVC (β = 7.80, 95% CI: 6.70 to 8.70), while asthma was negatively associated with FE1 (β = −255, 95% CI: −421 to −89) and FEV1/FVC (β = −0.04, 95% CI: −0.06 to −0.03). In the adjusted model (model 2), we found that the positive association of Se with FEV1 and FVC was still significant, and an inverse association among asthma, FEV1 and FEV1/FVC also existed. The adjusted model indicated that every 1-unit increase in Se intake, FEV1 and FVC increased 3.30 mL (95% CI: 2.60 to 4.00) and 4.30 mL (95% CI: 3.50 to 5.10), respectively. Furthermore, participants with current asthma had decreased lung function compared to those without current asthma (FEV1/FVC: β = −0.04, 95% CI: −0.06 to −0.02; FEV1: β = −215, 95% CI: −340 to −90). Multicollinearity was not present for all variables (variance inflation factor, VIF < 5) (Table 2).

**Table 2:** Associations among asthma, Se and lung function using general linear regression.

Outcomes	Model 1 (Unadjusted)			Model 2 (Adjusted)		
	β(95% CI)	*p* value	VIF	β(95% CI)	*p* value	VIF
FEV1/FVC						
Without current asthma	Reference	(−)		Reference	(−)	
With current asthma	−0.04 (−0.06, −0.03)	<0.001	1.02	−0.04 (−0.06, −0.02)	<0.001	2.06
Se	0.00 (0.00, 0.00)	0.600	1.020	0.00 (0.00, 0.00)	0.741	2.260
FEV1						
Without current asthma	Reference	(−)		Reference	(−)	
With current asthma	−255 (−421, −89)	0.002	1.01	−215 (−340, −90)	<0.001	3.14
Se	6.30 (5.50, 7.10)	<0.001	1.01	3.30 (2.60, 4.00)	<0.001	2.37
FVC						
Without current asthma	Reference	(−)		Reference	(−)	
With current asthma	−128 (−302, 46)	0.144	1.02	−69 (−203, 68)	0.312	3.12
Se	7.80 (6.70, 8.70)	<0.001	1.02	4.30 (3.50, 5.10)	<0.001	2.21

Covariates in model 2 included age, race, PIR, education levels, triglyceride, smoking status, BMI, ASBP, ADBP, urinary creatinine, high-density cholesterol (HDL), hemoglobin.

**Figure 3:**
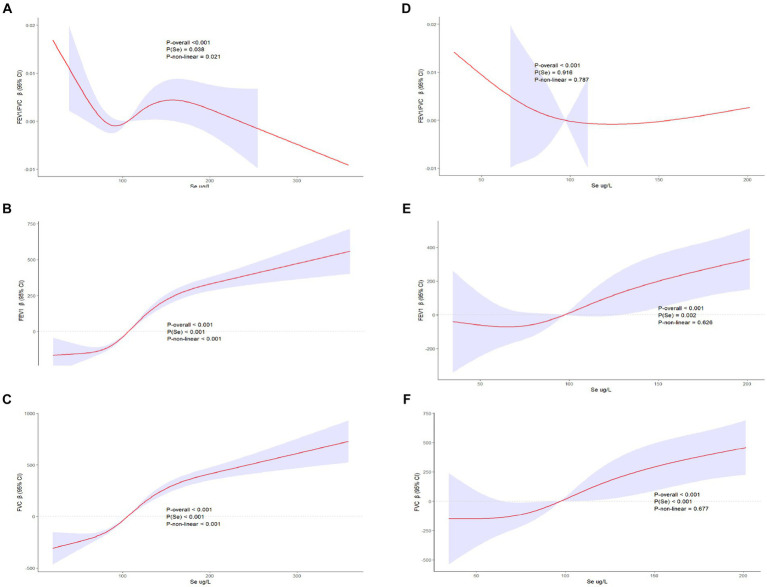
Restricted cubic spline of the association of asthma and lung function. **(A)** Asthma− and FEV1/FVC; **(B)** Asthma− and FEV1; **(C)** Asthma− and FVC; **(D)** Asthma+ and FEV1/FVC; **(E)** Asthma+ and FEV1; **(F)** Asthma+ and FVC; Red line and blue transparent area represent smooth curve fit and 95% CI fit, respectively. Adjusted for age, sex, race, PIR, education levels, triglycerides, smoking status, BMI, ASBP, ADBP, urinary creatinine, high-density cholesterol (HDL), and hemoglobin.

The restricted cubic spline visualized the associations among asthma, Se intake and lung function (Figure 3). In the asthma group (Figures 3A–C), the intake of selenium was correlated with FEV1, FVC, and FEV1/FVC (P for Se < 0.05), and this correlation was nonlinear (P-nonlinear <0.05). Se intake was positively correlated with FEV1 (Figure 3B) and FVC (Figure 3C) when it was approximately higher than 100 mcg. Moreover, this positive effect increased with increasing intake of Se. In contrast, when the intake of Se was approximately lower than 100 mcg, Se intake was negatively correlated with FEV1 (Figure 3B) and FVC (Figure 3C). This positive effect decreased with increasing intake of Se. In the asthma+ group, the intake of selenium was associated with FEV1 (Figure 3E) and FVC (Figure 3F) (P for Se < 0.05), but this association was linear (P-nonlinear >0.05). Similarly, Se intake was positively correlated with FEV1 (Figure 3E) and FVC (Figure 3F), when the intake of Se was approximately higher than 100 mcg.

### 3.4 Effects of different levels of selenium intake on lung function in participants with current asthma

**Figure 4:**
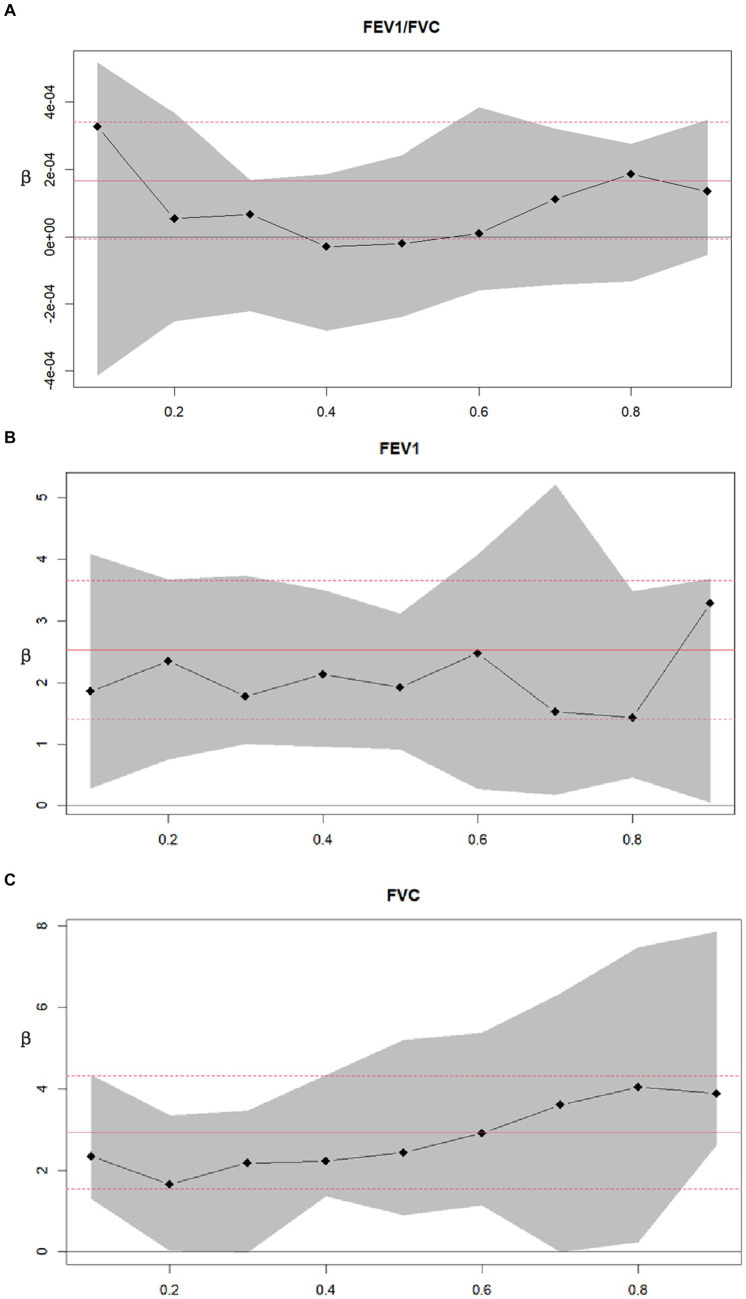
Decimal quantile regression of the association of asthma and lung function. **(A)** Asthma+ and FEV1/FVC; **(B)** Asthma+ and FEV1; **(C)** Asthma+ and FVC; Black line and gray transparent area represent smooth curve fit and 95% CI fit, respectively. Adjusted for age, sex, race, PIR, education levels, triglycerides, smoking status, BMI, ASBP, ADBP, urinary creatinine, high-density cholesterol (HDL), and hemoglobin.

**Figure 5:**
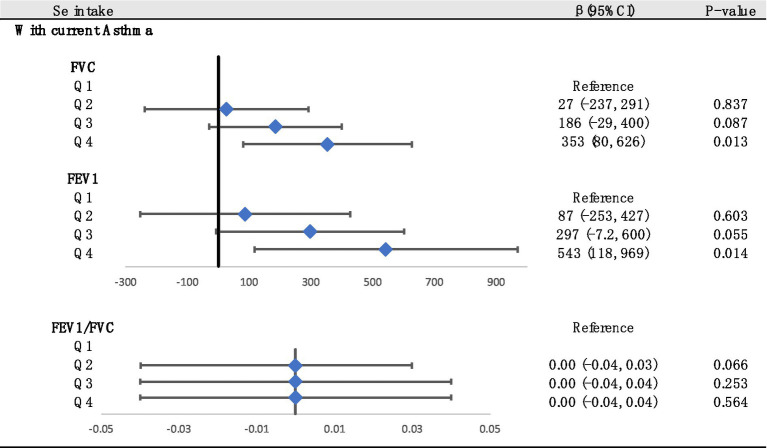
Associations between lung function and Se (divided into Q1, Q2, Q3 and Q4 according to quartiles, Q1: Se ≤ 76.75 mcg/d; Q2: 76.75–105.1 mcg/d; Q3: 105.1–137.65 mcg/d; Q4: Se ≥137.65 mcg/d). Adjusted for age, sex, race, PIR, education levels, triglycerides, smoking status, BMI, ASBP, ADBP, urinary creatinine, high-density cholesterol (HDL), and hemoglobin.

Decimal quantile regression was used to assess the effects of different levels of Se intake on lung function (Figure 4A). We found that in the asthma+ group, the positive effects of Se intake on FEV1 (Figure 4B) were significant for all percentiles and were rarely affected by the intake of Se. (The β of Se intake did not vary significantly with Se intake). However, the positive effects of Se intake on FVC (Figure 4C) were enhanced with the increase in Se intake and were significant for all percentiles except the 30th percentile and 70th percentile (the β of Se intake increased with increasing Se intake). The greatest positive effects were found in the 90th percentile of Se intake. We did not find any significant association between FEV1/FVC and Se intake for all percentiles. Next, to investigate the effects of different intakes of selenium on lung function in the asthma+ group, we classified selenium intake by quartile and performed multiple linear regression in the asthma+ group (Figure 5). Q1 was used as a reference. The FVC and FEV1 of participants with Q4 intake increased 353 mL (95% CI: 80 to 625) and 543 mL (95% CI: 118 to 969), respectively, relative to Q1 intake. Other results were not significant.

**Figure 6:**
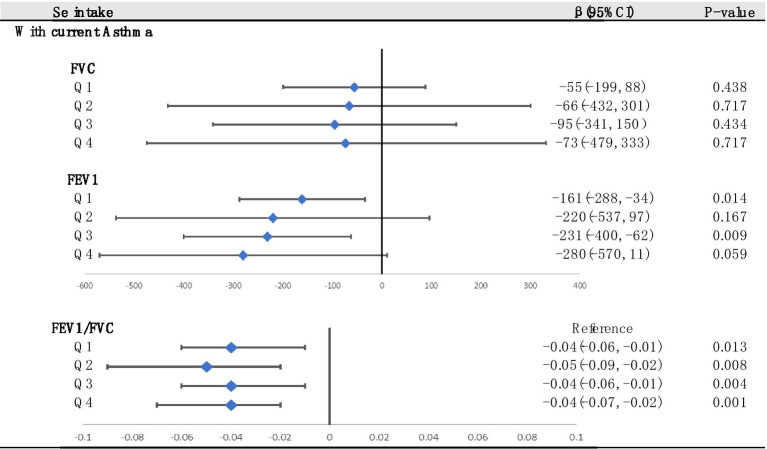
Stratified analysis of associations between asthma and lung function according to the quartiles of Se (divided into Q1, Q2, Q3 and Q4 according to quartiles, Q1: Se ≤ 76.75 mg; Q2: 76.75–105.1 mg; Q3: 105.1–137.65 mg; Q4: Se ≥137.65 mg). Adjusted for age, sex, race, PIR, education levels, triglycerides, smoking status, BMI, ASBP, ADBP, urinary creatinine, high-density cholesterol (HDL), and hemoglobin.

We used multiple linear regression to investigate whether the associations between asthma and lung function were still significant at different Se levels (Figure 6). The asthma group was used as a reference. We found that at the Q2 and Q4 levels, the correlation between FEV1 and asthma disappeared. In addition, asthma had the greatest adverse effect on FEV1/FVC at the Q2 level. In summary, maintaining Se intake at Q4 levels had the best beneficial impact on FEV1 and FVC.

### 3.5 Subgroup analysis

To verify whether the results of this study were robust, we conducted subgroup analysis based on demographic stratification (Table 3). Q4 levels of Se intake improved the FEV1 and FVC in participants older than 40 years. Similar results were observed in the male and non-Hispanic groups. This effect was not significant in the 20- to 40-year-old, female, Mexican American and other Hispanic groups. In other race groups, the data were not enough to analyze the relationship. No significant interaction was observed in any of the groups.

**Table 3:** Association between FEV1, FVC and Se intake in asthmatic participants.

Group		β (Q4 to Q1)	95%CI	*p* value	
Age					
20–40 (*N* = 132)	FEV1	155	(−190, 500)	0.38	
	FVC	142	(−260, 545)	0.49	
40–60 (*N* = 117)	FEV1	383	(29, 738)	0.037	**
	FVC	549	(108, 991)	0.017	**
60–80 and above (*N* = 80)	FEV1	524	(54, 993)	0.032	**
	FVC	715	(123, 1,306)	0.021	**
Interaction	FEV1	/	/	>0.05	
	FVC	/	/	>0.05	
Gender					
Male (*N* = 126)	FEV1	407	(−34, 849)	0.073	*
	FVC	579	(50, 1,108)	0.034	**
Female (*N* = 203)	FEV1	214	(−65, 492)	0.134	
	FVC	159	(−157, 475)	0.326	
Interaction	FEV1	/	/	>0.05	
	FVC	/	/	>0.05	
Race					
Non-Hispanic (*N* = 270)	FEV1	548	(300, 796)	<0.001	***
	FVC	669	(366, 972)	0.014	**
Mexican American (*N* = 24)	FEV1	354	(−221, 930)	0.256	
	FVC	452	(−316, 1,220)	0.276	
Other Hispanic (*N* = 22)	FEV1	−1826	(−4,396, 744)	0.201	
	FVC	−1818	(−4,696, 1,009)	0.243	
Other Race-Including Multi-Rucia (*N* = 13)		The data was not enough to analyze the relationship			
Interaction	FEV1	/	/	>0.05	
	FVC	/	/	>0.05	

The association among FEV1, FVC and Se intake in participants with current asthma.

Covariates included PIR, education levels, triglyceride, smoking status, BMI, ASBP, ADBP, urinary creatinine, high-density cholesterol (HDL), hemoglobin. *(0.05 < *p* < 0.1) **(0.01 < *p* < 0.05) ***(*p* < 0.01).

## 4 Discussion

Asthma is a recognized critical risk factor for lung health. This study undertook the validation of a negative association between asthma and lung function parameters, specifically FEV1 (forced expiratory volume in 1 s) and FEV1/FVC (FEV1 to forced vital capacity ratio). Additionally, our findings revealed a positive association between Se intake and lung function parameters (FEV1 and FVC). Furthermore, within the subgroup of asthmatic patients, our investigation demonstrated a dose-dependent positive effect of selenium intake on FVC, with a more gradual effect observed on FEV1. Notably, individuals with high selenium intake (exceeding 137.65 mcg) exhibited enhanced lung function in comparison to those with low selenium intake (less than 76.75 mcg). Maintaining selenium intake at the highest quartile level (exceeding 137.65 mcg) produced the most pronounced beneficial impact on FEV1 and FVC among asthmatic patients.

Our results exhibited robustness in the subset of participants aged over 40 years, males, and non-Hispanic individuals. However, it is essential to acknowledge that the limited sample size in the other three racial groups involved may render the results less credible. To provide additional context, two baseline tables, categorized by age (Supplementary Table S1) and gender (Supplementary Table S2), have been included. Notably, our analysis revealed a lower proportion of individuals reporting smoking (inclusive of past and current smokers) among women compared to men (Supplementary Table S2), and a similar trend was observed in participants under 40 years (Supplementary Table S1). It is worth noting that previous research has reported a link between smoking and oxidative stress in the lungs (26), which could account for the lack of statistical significance in certain subgroup analyses.

The relationship between asthma and Se is notably intricate. Numerous epidemiological studies have elucidated that asthma incidence, prevalence, or severity exhibits associations with reduced Se status (27–29). For instance, in a study comprising 165 participants, researchers investigated Se and zinc (Zn) concentrations in fingernails of asthmatic children (30). Among children in the highest quartile of Se and Zn concentrations, there was a substantial fivefold reduction in the prevalence ratio of asthma, while those in the lowest Se range displayed an almost 2.5-fold increase in the asthma prevalence ratio. Nonetheless, it is important to acknowledge that several studies have failed to establish a definitive connection between asthma and Se. A large-scale, multiregional study involving 588 participants (including 12 asthmatic patients) conducted within the Global Allergy and Asthma European Network (GA2LEN) analyzed asthma prevalence and severity data from 14 centers across Europe and reported no significant association between serum Se concentration and asthma levels (31). Furthermore, an experimental study revealed that increased Se intake may mitigate oxidative stress in the lungs while simultaneously enhancing immune responses to allergens by augmenting T helper (Th) responses in mice (32).

According to previous studies, Se may improve the lung function of asthmatic patients through several possible mechanisms. First, Th2 cells produce IL-4, IL-5, and IL-13, which promote allergic asthma (33), and higher Se intake skews differentiation toward Th1/Treg and away from Th2 phenotypes (34). Second, Se acts through antioxidant selenoproteins (especially through glutathione peroxidase (GPX), the best-known member of the selenoprotein family) that reduce oxidative stress in the lungs, as well as the organism in general, to reduce the burden of asthma-associated airway inflammation.

Although Se intake is positively correlated with pulmonary function in asthmatic patients, long-term exposure to high concentrations of environmental Se has been shown to be a high-risk factor for human health. Human Se overdose can lead to selenium poisoning (35, 36), which, although very rare, may cause amyotrophic lateral sclerosis (37) irrespective of race or ethnicity (38–40). Mechanistically, cellular exposure to high doses of selenium can lead to increased intracellular ROS, which are considered the primary mediators of selenium-induced cytotoxicity (41). Although Se is generally regarded as an essential factor for antioxidant enzyme production, chemically, it can react with essential thiol groups to form intramolecular disulfide bonds (S-Se), or cysteine residues residing in substrates (42) and indirectly generate ROS. The enhanced oxidative cellular environment may lead to DNA damage and genomic instability, initiating cell apoptosis (43, 44). Thus, oxidative stress related to selenium toxicity may result in impaired immune function, cytotoxicity, genotoxicity, and carcinogenic effects (45–47). Excessive selenium intake can be fatal and based on previous studies, an intake of less than 200 mcg/day is deemed appropriate (48).

First, it is worth noting that our study incorporated a substantial sample size, which is expected to enhance the reliability of our results. Furthermore, we conducted an in-depth investigation into the relationship between selenium and lung function in asthma patients across different levels of selenium intake, a relatively rare approach in prior research. Additionally, we examined the most effective selenium intake for improving lung function in asthma patients. Finally, we validated the stability of our results in various subgroups of the population and provided potential explanations for the lack of significant findings in certain groups. These comprehensive steps are expected to contribute to a more nuanced understanding of our academic research.

As a cross-sectional study, it is important to acknowledge that our results may not carry the same level of conviction as randomized controlled trials and cohort studies. Consequently, further randomized controlled trials and cohort studies may be necessary to elucidate the relationship between selenium (Se) intake and asthma more definitively. Additionally, we excluded a significant number of participants due to missing data, which could impact the credibility of our conclusions. It is worth noting that there exists a certain degree of data bias in the NHANES database, which could introduce interference with our experimental results. Finally, it should be acknowledged that some covariates influencing Se and asthma might not have been included in our model.

## 5 Conclusion

In summary, asthma exhibited a significant inverse association with lung function, while Se intake had a positive effect on lung function. In addition, we found that moderate Se could improve the lung function of asthmatic patients. Our findings suggest that we should increase the detection of pulmonary function in asthmatic patients and that supplementation with moderate doses of Se may help prevent lung function. Based on the findings of this study and previous research, we recommend that asthma patients consume 137.65 mcg to 200 mcg of selenium daily to improve pulmonary function while avoiding the adverse effects of selenium on the human body.

## Data availability statement

The original contributions presented in the study are included in the article/Supplementary material, further inquiries can be directed to the corresponding authors.

## Ethics statement

The studies involving humans were approved by NCHS Ethics Review Board, Continuation of Protocol #2005-06, Protocol #2011-17. The studies were conducted in accordance with the local legislation and institutional requirements. The participants provided their written informed consent to participate in this study. Written informed consent was obtained from the individual(s) for the publication of any potentially identifiable images or data included in this article.

## Author contributions

HJ: Conceptualization, Data curation, Methodology, Project administration, Software, Visualization, Writing – original draft, Writing – review & editing. GY: Conceptualization, Data curation, Investigation, Methodology, Project administration, Writing – review & editing, Writing – original draft. JinC: Data curation, Methodology, Project administration, Writing – original draft, Writing – review & editing. SY: Investigation, Project administration, Resources, Software, Writing – review & editing. JW: Investigation, Methodology, Project administration, Resources, Software, Supervision, Writing – review & editing. JZ: Resources, Software, Supervision, Validation, Visualization, Writing – review & editing. LZ: Project administration, Software, Supervision, Writing – review & editing. JY: Project administration, Software, Supervision, Writing – review & editing. JL: Data curation, Funding acquisition, Methodology, Project administration, Supervision, Writing – review & editing. JiaC: Data curation, Funding acquisition, Supervision, Writing – review & editing. YY: Conceptualization, Formal analysis, Funding acquisition, Project administration, Supervision, Writing – review & editing.

## Supplementary material

SUPPLEMENTARY TABLE S1 Baseline characteristics stratified by age.

SUPPLEMENTARY TABLE S2 Baseline characteristics stratified by sex.

## References

[1] Dharmage SC Perret JL Custovic A. Epidemiology of asthma in children and adults. Front Pediatr. (2019) 7:246. doi: 10.3389/fped.2019.00246, PMID: 31275909 PMC6591438

[2] Chung KF Marwick JA. Molecular mechanisms of oxidative stress in airways and lungs with reference to asthma and chronic obstructive pulmonary disease. Ann N Y Acad Sci. (2010) 1203:85–91. doi: 10.1111/j.1749-6632.2010.05600.x, PMID: 20716288

[3] Michaeloudes C Abubakar-Waziri H Lakhdar R Raby K Dixey P Adcock IM. Molecular mechanisms of oxidative stress in asthma. Mol Asp Med. (2022) 85:101026. doi: 10.1016/j.mam.2021.101026 34625291

[4] Teng Y Sun P Zhang J Yu R Bai J Yao X. Hydrogen peroxide in exhaled breath condensate in patients with asthma: a promising biomarker? Chest. (2011) 140:108–16. doi: 10.1378/chest.10-2816, PMID: 21436249

[5] Ganas K Loukides S Papatheodorou G Panagou P Kalogeropoulos N. Total nitrite/nitrate in expired breath condensate of patients with asthma. Respir Med. (2001) 95:649–54. doi: 10.1053/rmed.2001.1117, PMID: 11530952

[6] Nadeem A Chhabra SK Masood A Raj HG. Increased oxidative stress and altered levels of antioxidants in asthma. J Allergy Clin Immunol. (2003) 111:72–8. doi: 10.1067/mai.2003.17 12532099

[7] Ozaras R Tahan V Turkmen S Talay F Besirli K Aydin S. Changes in malondialdehyde levels in bronchoalveolar fluid and serum by the treatment of asthma with inhaled steroid and beta2-agonist. Respirology. (2000) 5:289–92. doi: 10.1046/j.1440-1843.2000.00260.x, PMID: 11022993

[8] Karadogan B Beyaz S Gelincik A Buyukozturk S Arda N. Evaluation of oxidative stress biomarkers and antioxidant parameters in allergic asthma patients with different level of asthma control. J Asthma. (2022) 59:663–72. doi: 10.1080/02770903.2020.1870129, PMID: 33380228

[9] Steinbrenner H Sies H. Protection against reactive oxygen species by selenoproteins. Biochim Biophys Acta. (2009) 1790:1478–85. doi: 10.1016/j.bbagen.2009.02.014 19268692

[10] Steinbrenner H Speckmann B Klotz LO. Selenoproteins: Antioxidant selenoenzymes and beyond. Arch Biochem Biophys. (2016) 595:113–9. doi: 10.1016/j.abb.2015.06.024, PMID: 27095226

[11] Brigelius-Flohé R Flohé L. Selenium and redox signaling. Arch Biochem Biophys. (2017) 617:48–59. doi: 10.1016/j.abb.2016.08.003 27495740

[12] Huang Z Rose AH Hoffmann PR. The role of selenium in inflammation and immunity: from molecular mechanisms to therapeutic opportunities. Antioxid Redox Signal. (2012) 16:705–43. doi: 10.1089/ars.2011.4145, PMID: 21955027 PMC3277928

[13] Ventura M Melo M Carrilho F. Selenium and thyroid disease: from pathophysiology to treatment. Int J Endocrinol. (2017) 2017:1297658–9. doi: 10.1155/2017/1297658, PMID: 28255299 PMC5307254

[14] Schomburg L. Selenium, selenoproteins and the thyroid gland: interactions in health and disease. Nat Rev Endocrinol. (2011) 8:160–71. doi: 10.1038/nrendo.2011.174, PMID: 22009156

[15] Jones GD Droz B Greve P Gottschalk P Poffet D McGrath SP. Selenium deficiency risk predicted to increase under future climate change. Proc Natl Acad Sci USA. (2017) 114:2848–53. doi: 10.1073/pnas.1611576114, PMID: 28223487 PMC5358348

[16] Vinceti M Crespi CM Malagoli C Del Giovane C Krogh V. Friend or foe? The current epidemiologic evidence on selenium and human cancer risk. J Environ Sci Health C Environ Carcinog Ecotoxicol Rev. (2013) 31:305–41. doi: 10.1080/10590501.2013.844757, PMID: 24171437 PMC3827666

[17] Rita Cardoso B Silva Bandeira V Jacob-Filho W Franciscato Cozzolino SM. Selenium status in elderly: relation to cognitive decline. J Trace Elem Med Biol. (2014) 28:422–6. doi: 10.1016/j.jtemb.2014.08.009, PMID: 25220532

[18] Cardoso BR Hare DJ Bush AI Li QX Fowler CJ Masters CL. Selenium levels in serum, red blood cells, and cerebrospinal fluid of Alzheimer's disease patients: a report from the Australian imaging, Biomarker & Lifestyle Flagship Study of ageing (AIBL). J Alzheimers Dis. (2017) 57:183–93. doi: 10.3233/JAD-160622, PMID: 28222503

[19] Cardoso BR Roberts BR Bush AI Hare DJ. Selenium, selenoproteins and neurodegenerative diseases. Metallomics. (2015) 7:1213–28. doi: 10.1039/c5mt00075k, PMID: 25996565

[20] de Farias CR Cardoso BR de Oliveira GM de Mello Guazzelli IC Catarino RM Chammas MC. A randomized-controlled, double-blind study of the impact of selenium supplementation on thyroid autoimmunity and inflammation with focus on the GPx1 genotypes. J Endocrinol Investig. (2015) 38:1065–74. doi: 10.1007/s40618-015-0285-8, PMID: 25894865

[21] Köhrle J. Selenium and the thyroid. Curr Opin Endocrinol Diabetes Obes. (2015) 22:392–401. doi: 10.1097/MED.0000000000000190 26313901

[22] Fu YX Wang YB Bu QW Guo MY. Selenium deficiency caused fibrosis as an oxidative stress-induced inflammatory injury in the lungs of mice. Biol Trace Elem Res. (2023) 201:1286–300. doi: 10.1007/s12011-022-03222-6, PMID: 35397105

[23] Mu J Lei L Zheng Y Liu J Li J Li D. Oxidative stress induced by selenium deficiency contributes to inflammation, apoptosis and necroptosis in the lungs of calves. Antioxidants (Basel). (2023) 12:796. doi: 10.3390/antiox12040796, PMID: 37107171 PMC10135166

[24] Jiang J Mehrabi Nasab E Athari SM Athari SS. Effects of vitamin E and selenium on allergic rhinitis and asthma pathophysiology. Respir Physiol Neurobiol. (2021) 286:103614. doi: 10.1016/j.resp.2020.103614, PMID: 33422684

[25] Fan H Xiong Y Huang Y Wang L Xu C Li W. Moderate selenium alleviates the pulmonary function impairment induced by cadmium and lead in adults: a population-based study. Sci Total Environ. (2023) 903:166234. doi: 10.1016/j.scitotenv.2023.166234, PMID: 37572899

[26] Pryor WA Prier DG Church DF. Electron-spin resonance study of mainstream and sidestream cigarette smoke: nature of the free radicals in gas-phase smoke and in cigarette tar. Environ Health Perspect. (1983) 47:345–55. doi: 10.1289/ehp.8347345, PMID: 6297881 PMC1569403

[27] de Luis DA Izaola O Aller R Armentia A Cuéllar L. Ingesta de antioxidantes y grasas en pacientes con asma polínica [Antioxidant and fat intake in patients with polinic asthma]. Med Clin (Barc). (2003) 121:653–4. doi: 10.1016/S0025-7753(03)74050-0, PMID: 14642226

[28] Qujeq D Hidari B Bijani K Shirdel H. Glutathione peroxidase activity and serum selenium concentration in intrinsic asthmatic patients. Clin Chem Lab Med. (2003) 41:200–2. doi: 10.1515/CCLM.2003.032, PMID: 12667007

[29] Omland Ø Deguchi Y Sigsgaard T Hansen JC. Selenium serum and urine is associated with mild asthma and atopy. The SUS study. J Trace Elem Med Biol. (2002) 16:123–7. doi: 10.1016/S0946-672X(02)80039-6, PMID: 12195727

[30] Carneiro MF Rhoden CR Amantéa SL Barbosa F Jr. Low concentrations of selenium and zinc in nails are associated with childhood asthma. Biol Trace Elem Res. (2011) 144:244–52. doi: 10.1007/s12011-011-9080-3, PMID: 21607705

[31] Burney P Potts J Makowska J Kowalski M Phillips J Gnatiuc L. A case–control study of the relation between plasma selenium and asthma in European populations: a GAL2EN project. Allergy. (2008) 63:865–71. doi: 10.1111/j.1398-9995.2008.01716.x, PMID: 18588552

[32] Hoffmann PR Jourdan-Le Saux C Hoffmann FW Chang PS Bollt O He Q. A role for dietary selenium and selenoproteins in allergic airway inflammation. J Immunol. (2007) 179:3258–67. doi: 10.4049/jimmunol.179.5.3258, PMID: 17709542

[33] Norton RL Hoffmann PR. Selenium and asthma. Mol Asp Med. (2012) 33:98–106. doi: 10.1016/j.mam.2011.10.003, PMID: 22024250 PMC3246085

[34] Hoffmann FW Hashimoto AC Shafer LA Dow S Berry MJ Hoffmann PR. Dietary selenium modulates activation and differentiation of CD4+ T cells in mice through a mechanism involving cellular free thiols. J Nutr. (2010) 140:1155–61. doi: 10.3945/jn.109.120725, PMID: 20375261 PMC2869499

[35] Lenz M Lens PN. The essential toxin: the changing perception of selenium in environmental sciences. Sci Total Environ. (2009) 407:3620–33. doi: 10.1016/j.scitotenv.2008.07.056, PMID: 18817944

[36] Lee KH Jeong D. Bimodal actions of selenium essential for antioxidant and toxic pro-oxidant activities: the selenium paradox (review). Mol Med Rep. (2012) 5:299–304. doi: 10.3892/mmr.2011.651, PMID: 22051937

[37] Estevez AO Morgan KL Szewczyk NJ Gems D Estevez M. The neurodegenerative effects of selenium are inhibited by FOXO and PINK1/PTEN regulation of insulin/insulin-like growth factor signaling in *Caenorhabditis elegans*. Neurotoxicology. (2014) 41:28–43. doi: 10.1016/j.neuro.2013.12.012, PMID: 24406377 PMC3979119

[38] Roman M Jitaru P Barbante C. Selenium biochemistry and its role for human health. Metallomics. (2014) 6:25–54. doi: 10.1039/c3mt00185g 24185753

[39] Vinceti M Solovyev N Mandrioli J Crespi CM Bonvicini F Arcolin E. Cerebrospinal fluid of newly diagnosed amyotrophic lateral sclerosis patients exhibits abnormal levels of selenium species including elevated selenite. Neurotoxicology. (2013) 38:25–32. doi: 10.1016/j.neuro.2013.05.016, PMID: 23732511 PMC3770807

[40] Schomburg L. Dietary selenium and human health. Nutrients. (2016) 9:22. doi: 10.3390/nu9010022, PMID: 28042811 PMC5295066

[41] Sun J Zheng X Li H Fan D Song Z Ma H. Monodisperse selenium-substituted hydroxyapatite: controllable synthesis and biocompatibility. Mater Sci Eng C Mater Biol Appl. (2017) 73:596–602. doi: 10.1016/j.msec.2016.12.106, PMID: 28183650

[42] Ganther HE. Selenium metabolism, selenoproteins and mechanisms of cancer prevention: complexities with thioredoxin reductase. Carcinogenesis. (1999) 20:1657–66. doi: 10.1093/carcin/20.9.1657, PMID: 10469608

[43] Chung YW Kim TS Lee SY Lee SH Choi Y Kim N. Selenite-induced apoptosis of osteoclasts mediated by the mitochondrial pathway. Toxicol Lett. (2006) 160:143–50. doi: 10.1016/j.toxlet.2005.06.019, PMID: 16111838

[44] Zhu Z Jiang W Ganther HE Ip C Thompson HJ. In vitro effects of se-allylselenocysteine and se-propylselenocysteine on cell growth, DNA integrity, and apoptosis. Biochem Pharmacol. (2000) 60:1467–73. doi: 10.1016/s0006-2952(00)00461-5, PMID: 11020448

[45] Mostofa MG Hossain MA Siddiqui MN Fujita M Tran LS. Phenotypical, physiological and biochemical analyses provide insight into selenium-induced phytotoxicity in rice plants. Chemosphere. (2017) 178:212–23. doi: 10.1016/j.chemosphere.2017.03.046, PMID: 28324842

[46] Prasad KS Selvaraj K. Biogenic synthesis of selenium nanoparticles and their effect on as(III)-induced toxicity on human lymphocytes. Biol Trace Elem Res. (2014) 157:275–83. doi: 10.1007/s12011-014-9891-0, PMID: 24469678

[47] Selvaraj V Yeager-Armstead M Murray E. Protective and antioxidant role of selenium on arsenic trioxide-induced oxidative stress and genotoxicity in the fish hepatoma cell line PLHC-1. Environ Toxicol Chem. (2012) 31:2861–9. doi: 10.1002/etc.2022, PMID: 23023949

[48] Valenzuela R Das UN Videla LA Llorente CG. Nutrients and diet: a relationship between oxidative stress, aging, obesity, and related noncommunicable diseases. Oxidative Med Cell Longev. (2018) 2018:7460453. doi: 10.1155/2018/7460453, PMID: 30116490 PMC6079337

