# Supplementary material for: The correlation between selenium intake and lung function in asthmatic people: a cross-sectional study
Source: Front Nutr. 2024 May 17;11:1362119. doi: 10.3389/fnut.2024.1362119 (PMC11141543; doi:10.3389/fnut.2024.1362119)
Supplement: SUPPLEMENTARY TABLE S1 — Baseline characteristics stratified by age. [file Table_1.docx]

**Table S1** Baseline characteristics stratified by age.

**Table S2** Baseline characteristics stratified by gender.
